# Therapeutic effect of laparoscopic salpingotomy vs. salpingectomy on patients with ectopic pregnancy: A systematic review and meta-analysis

**DOI:** 10.3389/fsurg.2022.997490

**Published:** 2022-10-11

**Authors:** Lin Wenjing, Li Haibo

**Affiliations:** Department of Obstetrics, Baoji Central Hospital, Baoji, China

**Keywords:** tubal pregnancy, salpingotomy, salpingectomy, meta-analysis, RCT (randomised controlled trial)

## Abstract

**Background and aim:**

Laparoscopic treatment of ectopic pregnancy mainly includes laparoscopic salpingotomy and salpingectomy. We aimed to assess the therapeutic effect of laparoscopic salpingotomy and salpingectomy on patients with tubal pregnancy.

**Methods:**

From January 2000 through June 2022, the Cochrane Library, Medline, PubMed, Web of Science, EMBASE, and the Chinese Biomedicine Database were searched for studies that compared the therapeutic effect of laparoscopic salpingotomy vs. salpingectomy in the treatment of tubal pregnancy.

**Results:**

Twenty-four randomized clinical trials (RCTs) studies were incorporated into this analysis. No statistical differences were found between the two groups in terms of operation duration and postoperative hospitalize length, but the volume of intraoperative blood loss in patients with laparoscopic salpingotomy was less than that in salpingectomy. Importantly, the natural intrauterine pregnancy rate after laparoscopic salpingotomy was significantly higher than those who underwent salpingectomy. In addition, laparoscopic salpingotomy can better protect the ovarian reserve function and endocrine function and provide favorable conditions for the second pregnancy.

**Conclusion:**

Patients with ectopic tubal pregnancy should give priority to laparoscopic salpingotomy for embryo extraction.

## Introduction

Ectopic pregnancy refers to the implantation of fertilized eggs outside the normal site and is one of the common gynecological diseases (1). The fallopian tube is the most common site of ectopic pregnancy (about 95%) (2). If the patient does not receive treatment in time, once the fallopian tube ruptures due to embryonic development, it can lead to massive bleeding in the abdominal cavity of the patient and, in serious cases, can endanger the life of the patient. For patients with ectopic pregnancy, the embryo must be removed from the fallopian tube to terminate the pregnancy. At present, surgery is the most effective way to treat ectopic pregnancy (3–5).

Laparoscopic fallopian tube surgery only has small trauma and rapid postoperative recovery; hence, it should be the first choice for the treatment of fallopian tube pregnancy (6–8). Laparoscopic treatment of ectopic pregnancy mainly includes laparoscopic salpingotomy and salpingectomy. Although both methods have good curative effects, the effects of different surgical methods on the postoperative reproductive function of patients have not yielded a consistent conclusion. Fernandez et al. (9) found that the two-year intrauterine pregnancy rate in the salpingotomy group was significantly higher than that in the salpingectomy group and believed that salpingotomy had a greater advantage in the postoperative intrauterine pregnancy rate. On the contrary, Mol et al. (10) conducted a multicenter, randomized controlled study and found no significant difference in the postoperative intrauterine pregnancy rate and the re-ectopic pregnancy rate between the two groups. Besides, there have been relatively few clinical studies on the effects of different laparoscopic methods for surgical treatment of tubal pregnancy on ovarian blood supply and ovarian reserve function, and the effects of the two surgical methods on patients' postoperative ovarian reserve function are still controversial.

With the emergence of new clinical research evidence in recent years, we conducted a meta-analysis to study the therapeutic effect of salpingotomy and salpingectomy on patients with tubal pregnancy. The perioperative safety, postoperative natural intrauterine pregnancy rate, ectopic pregnancy rate of the two surgical methods, and the effects of the two surgical methods on the ovarian reserve function of patients were compared.

## Methods

### Search strategies

**Figure 1:**
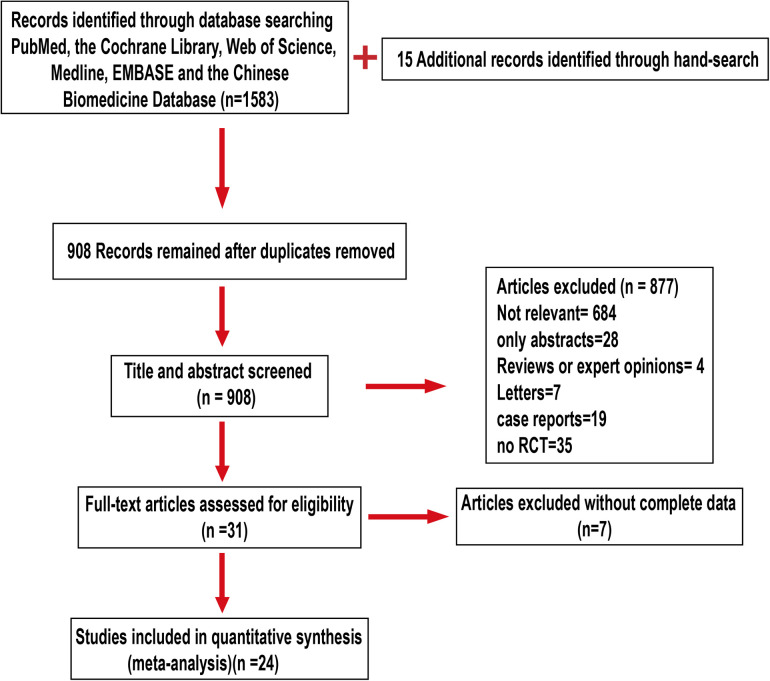
Flow diagram.

From January 2000 through June 2022, the Cochrane Library, Medline, PubMed, Web of Science, EMBASE, and the Chinese Biomedicine Database were searched. Our subject-related searches comprised both free and MESH terms without language constraints. The following keywords were used in the search: (“tubal pregnancy” OR “ectopic pregnancy” OR “tubal ectopic pregnancy” OR “tubal gestation” OR “tubal ectopic pregnancy”) AND (“conservative surgery” OR “conservative procedure” OR “salpingotomy” OR “salpingostomy”) AND (“salpingectomy” OR “tubal excision” OR “tubectomy” OR “radical surgery” OR “radical procedure”) NOT “animals.” Two independent researchers used these keywords to search for titles, abstracts, and medical subject headings. The consensus was developed between the two researchers to make a final determination on the eligibility of the article for inclusion. When no consensus could be established, a third researcher made the ultimate decision. The outcomes of the investigated search approach are displayed as a flow diagram (Figure 1). Since the analysis was based on studies that were previously published, neither ethical approval nor patient permission was necessary.

### Inclusion and exclusion criteria

The studies included in this analysis were chosen based on the following criteria: (1) Studies including comparisons of groups of patients who underwent salpingectomy against salpingostomy; (2) Pregnancies resulting from *in vitro* fertilization were likewise excluded from all included studies; (3) If the same data had been published many times, only the most recent report was considered; (4) Only studies with at least one significant study result were considered for inclusion; (5) Only studies for which full-text access was accessible were considered; (6) RCT studies.

The studies listed below were eliminated from consideration in this study. (1) Studies that did not mention in detail the type of surgery that was performed; (2) Studies that do not have data readily available for extraction; (3) Analyses of non-tubal ectopic pregnancies; (4) Not RCT studies. (5) Studies are categorized as abstracts, case reports, reviews, letters, comments, animal studies or investigations with insufficient data.

### Data extraction and deﬁnition

The following outcome indices were used: (1) the volume of bleeding during operation; (2) operation duration; (3) postoperative hospitalize length; (4) HCG level: Serum HCG level was detected 12 h after the operation. (5). About 93% of second pregnancies occurred about 18 months after the operation (11), and only the data of second pregnancies from included literature with more than 18 months were analyzed. Intrauterine pregnancy: spontaneous intrauterine pregnancy during postoperative follow-up, including full-term delivery and spontaneous intrauterine pregnancy abortion. Ectopic pregnancy: ectopic pregnancy in any part that occurs again during postoperative follow-up. (6) Serum ovarian hormone level: The level of FSH (follicle-stimulating hormone), LH (luteinizing hormone), E2 (estradiol), T (testosterone) and P (progesterone) were detected 6 months after operation; (7). Ultrasound examination: EDV (end-diastolic velocity), PSV (peak systolic velocity), RI (resistance index), numbers of follicles, and cross-sectional area of the affected ovary were examined by ultrasound 6 months after the operation.

### Assessing the quality and the bias risk of included RCTs

**Figure 2:**
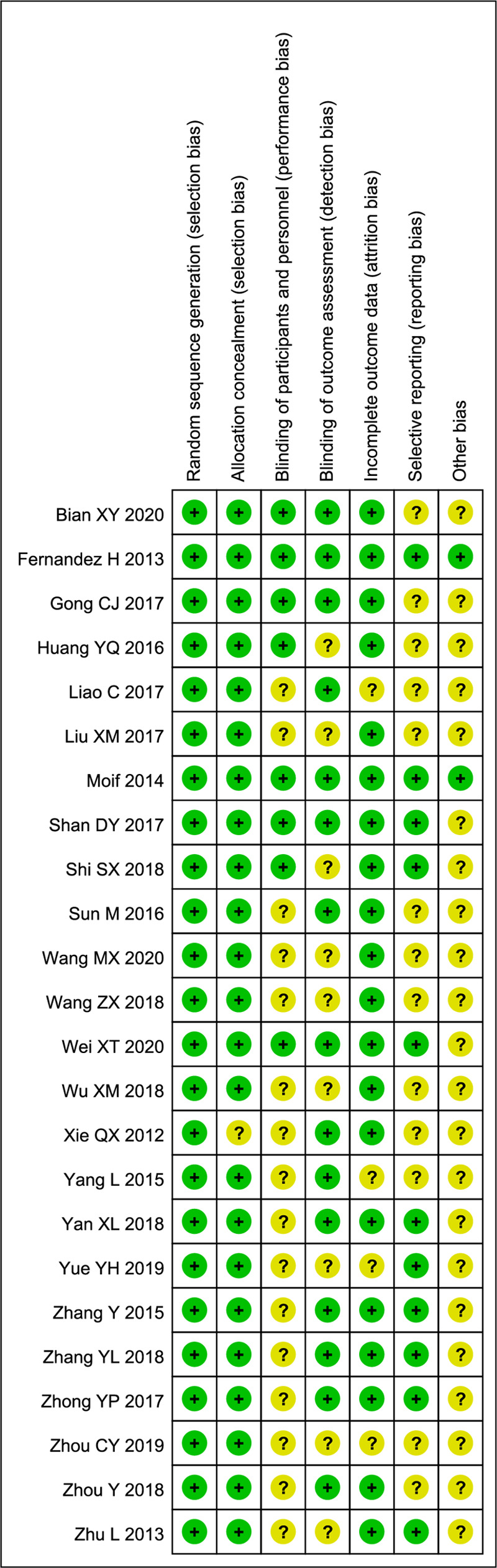
Risk of bias: “+” and “?” indicated the low risk and unclear risk of bias, respectively.

The methodological quality was assessed employing the modified Jadad scoring system. The risk of bias in the considered RCTs was assessed using the quality criteria recommended by the Cochrane Handbook (Figure 2).

## Statistical analysis

The Review Manager software was utilized to conduct meta-analyses. Both continuous and categorical variables were evaluated using weighted mean differences (WMDs) and odds ratios (ORs) with corresponding 95% CIs. Chi-squared, Mantel-Hansel, and I^2^ tests were employed to analyze to examine study heterogeneity. In instances where *I*^2^ was greater than or equal to 50%, a random-effects model was utilized, while if *I*^2^ less than 50%, a fixed-effects model was utilized. The funnel plot was applied to assess the publication bias. Differences with *p *< 0.05 were considered statistically significant.

## Results

### Quality and characteristics of the included RCT studies

This meta-analysis comprised a total of twenty-four RCTs. There were a total of 2,354 patients involved in the study, with 1,171 patients assigned to the salpingotomy group and 1,183 patients assigned to the salpingectomy group. Table 1 summarizes the parameters of the investigations that were incorporated into the meta-analysis. We determined the scores for each of the examined studies. The scores ranged from 4 to 7, suggesting that the included RCT studies were of good quality (Table 2).

**Table 1:** Main characteristics of all the included RCT studies.

Author	Year	Country	Group	N	Age, y	Outcome
Xie et al. (12)	2012	China	Salpingotomy	20	20–40	6,7
			Salpingectomy	20	20–40	
Fernandez et al. (9)	2013	Europe	Salpingotomy	101	29.28 (Mean)	5
			Salpingectomy	98	31.25 (Mean)	
Zhu et al. (13)	2013	China	Salpingotomy	20	25–35	6,7
			Salpingectomy	20	25–35	
Mol et al. (10)	2014	France	Salpingotomy	215	30.9 ± 5.5	5
			Salpingectomy	231	30.9 ± 5.5	
Yang et al. (14)	2015	China	Salpingotomy	34	21–37	6
			Salpingectomy	34	21–37	
Zhang et al. (15)	2015	China	Salpingotomy	30	29.0 ± 2.2	1,2,3,5,7
			Salpingectomy	30	32.0 ± 2.4	
Sun et al. (16)	2016	China	Salpingotomy	50	26.21 ± 3.26	1,2,3,4,5
			Salpingectomy	50	27.05 ± 3.12	
Huang et al. (17)	2016	China	Salpingotomy	35	27.5 ± 5.0	1,2,3,4,5
			Salpingectomy	35	26.1 ± 5.4	
Zhong et al. (18)	2017	China	Salpingotomy	42	27.49 ± 3.19	1,2,3,4,5
			Salpingectomy	42	27.75 ± 3.21	
Shan et al. (19)	2017	China	Salpingotomy	30	27.6 ± 6.3	6,7
			Salpingectomy	30	26.7 ± 6.9	
Gong et al. (20)	2017	China	Salpingotomy	36	35.7 ± 3.3	1,2,3,4
			Salpingectomy	36	37.1 ± 4.9	
Liao et al. (21)	2017	China	Salpingotomy	25	28.0 ± 5.0	6,7
			Salpingectomy	25	29.5 ± 5.5	
Liu et al. (22)	2017	China	Salpingotomy	50	28.2 ± 3.4	1,2,3,4,5
			Salpingectomy	50	27.9 ± 3.6	
Shi et al. (23)	2018	China	Salpingotomy	40	28.7 ± 3.4	5,6,7
			Salpingectomy	40	28.3 ± 3.6	
Wu et al. (24)	2018	China	Salpingotomy	30	20–40	6
			Salpingectomy	30	20–40	
Wang et al. (25)	2018	China	Salpingotomy	60	29.87 ± 4.56	5
			Salpingectomy	60	28.47 ± 4.38	
Yan et al. (26)	2018	China	Salpingotomy	40	29.54 ± 3.74	1,2,5
			Salpingectomy	40	29.57 ± 3.80	
Zhang et al. (27)	2018	China	Salpingotomy	38	28.48 (Mean)	1,2,3,4,5
			Salpingectomy	38	28.37 (Mean)	
Zhou et al. (28)	2018	China	Salpingotomy	53	27.83 ± 1.16	5
			Salpingectomy	53	27.83 ± 1.16	
Yue et al. (29)	2019	China	Salpingotomy	40	28.27 ± 4.43	5,6
			Salpingectomy	39	29.49 ± 5.13	
Zhou et al. (30)	2019	China	Salpingotomy	30	29.47 ± 4.35	5,6,7
			Salpingectomy	30	28.86 ± 4.15	
Wei et al. (31)	2020	China	Salpingotomy	54	29.54 ± 2.36	1,2,3,5
			Salpingectomy	54	28.43 ± 3.21	
Wang et al. (32)	2020	China	Salpingotomy	40	25.84 ± 3.11	2,4,5
			Salpingectomy	40	26.78 ± 3.58	
Bian et al. (33)	2020	China	Salpingotomy	58	28.75 ± 4.2	2,5
			Salpingectomy	58	27.9 ± 4.55	

Outcome: 1: Time of operation; 2: intraoperative blood loss; 3: postoperative hospital stay; 4: HCG level; 5: Intrauterine pregnancy and ectopic pregnancy; 6: Serum ovarian hormone level; 7: Ultrasound examination.

**Table 2:** Modified Jadad scale system for randomized controlled trials.

Refs	A	B	C	D	E
Xie et al. (12)	2	1	1	0	4
Fernandez et al. (9)	2	2	2	1	7
Zhu et al. (13)	2	1	1	0	4
Mol et al. (10)	2	2	2	1	7
Yang et al. (14)	2	2	1	0	5
Zhang et al. (15)	2	1	1	0	4
Sun et al. (16)	2	1	1	0	4
Huang et al. (17)	2	2	1	0	5
Zhong et al. (18)	2	2	1	1	6
Shan et al. (19)	2	2	1	1	6
Gong et al. (20)	2	2	1	1	6
Liao et al. (21)	2	2	1	0	5
Liu et al. (22)	2	2	0	0	4
Shi et al. (23)	2	2	1	0	5
Wu et al. (24)	2	2	0	0	4
Wang et al. (25)	2	2	0	0	4
Yan et al. (26)	2	2	1	0	5
Zhang et al. (27)	2	2	1	1	6
Zhou et al. (28)	2	2	1	0	5
Yue et al. (29)	2	2	0	0	4
Zhou et al. (30)	2	2	1	0	5
Wei et al. (31)	2	2	1	1	6
Wang et al. (32)	2	2	0	0	4
Bian et al. (33)	2	2	1	1	6

1. Modified Jadad scale system: The system was used to assess randomization, concealment of allocation, blinding, and withdrawals in the study. Each item was given a score of 0–2 and 7 score in total. If the total score was ≥4, the RCT was of high quality.

2. A: Randomization; B: Concealment of allocation; C: Double blinding; D: Withdrawals and drop out; E: Total Score.

### Meta-analysis of perioperative safety

#### Operating duration

**Figure 3:**
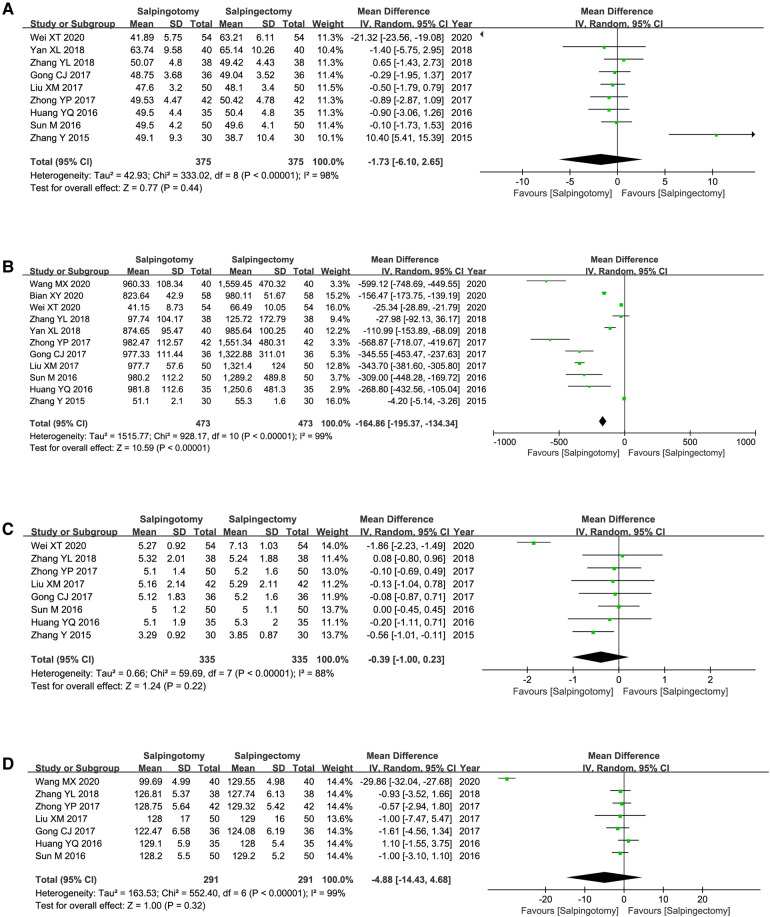
Meta-analysis of the perioperative safety. (**A**) Operating duration; (**B**) Intraoperative blood loss; (**C**) Postoperative hospitalize length; (**D**) Postoperative hCG level.

Operation duration was reported by nine of the included studies. The results of the meta-analysis, which used a random model (*I*^2 ^= 98%), showed that there was no marked difference between two groups (WMD = −1.73; 95%CI, −6.10–2.65; *p *= 0.44) (Figure 3A).

#### Volume of bleeding during operation

Eleven included studies reported the volume of bleeding during operation. The results of the meta-analysis, which used a random model (*I*^2 ^= 99%), showed that the volume of bleeding was signiﬁcantly less in salpingotomy group (WMD = −164.86; 95%CI, −195.3–134.34; *p *< 0.00001) (Figure 3B).

#### Postoperative hospitalize length

Postoperative hospitalize length was reported by eight of the included studies. The results of the meta-analysis, which used a random model (*I*^2 ^= 88%), showed that there was no distinct difference between two groups (WMD = −0.39; 95%CI, −1.00–0.23; *p *= 0.22) (Figure 3C).

#### Postoperative hCG level

Postoperative hCG level was reported by seven of the included studies. The results of the meta-analysis, which used a random model (*I*^2 ^= 99%), showed that there was no distinct difference between two groups (WMD = −4.88; 95%CI, −14.43–4.68; *p *= 0.32) (Figure 3D).

### Meta-analysis of reproductive outcome after operation

#### Postoperative intrauterine pregnancy rate

**Figure 4:**
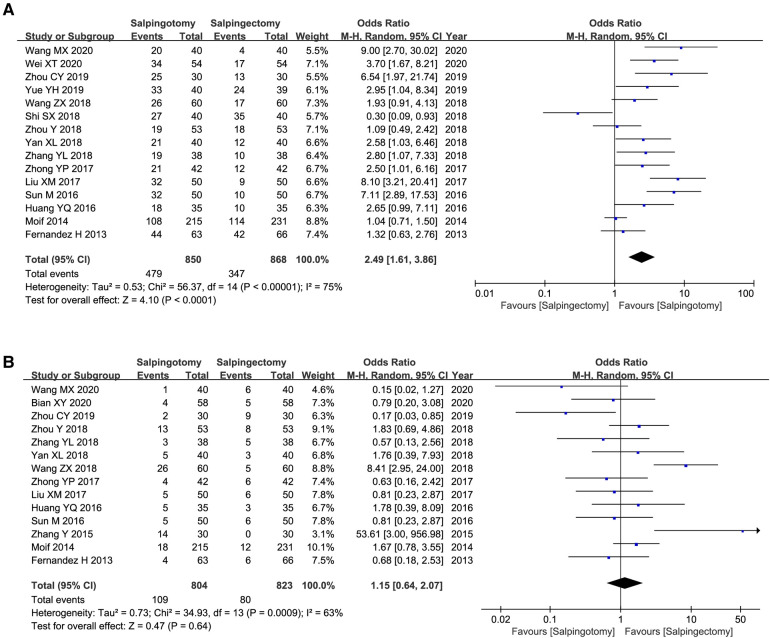
Meta-analysis of reproductive outcome after operation. (**A**) the rate of intrauterine pregnancy; (**B**) the rate of ectopic pregnancy rate.

Fifteen included studies reported the intrauterine pregnancy rate. The results of the meta-analysis, which used a random model (*I*^2 ^= 75%), showed that intrauterine pregnancy rate was signiﬁcantly higher in salpingotomy group (OR = 2.49; 95%CI, 1.61–3.86; *p *< 0.0001) (Figure 4A).

#### Postoperative ectopic pregnancy rate

Fourteen included studies reported the ectopic pregnancy rate. The results of the meta-analysis, which used a random model (*I*^2 ^= 63%), showed that there was no marked difference between two groups (OR = 1.15; 95%CI, 0.64–2.07; *p *= 0.64) (Figure 4B).

### Meta-analysis of ovarian function after the operation

#### Serum ovarian hormone level

**Figure 5:**
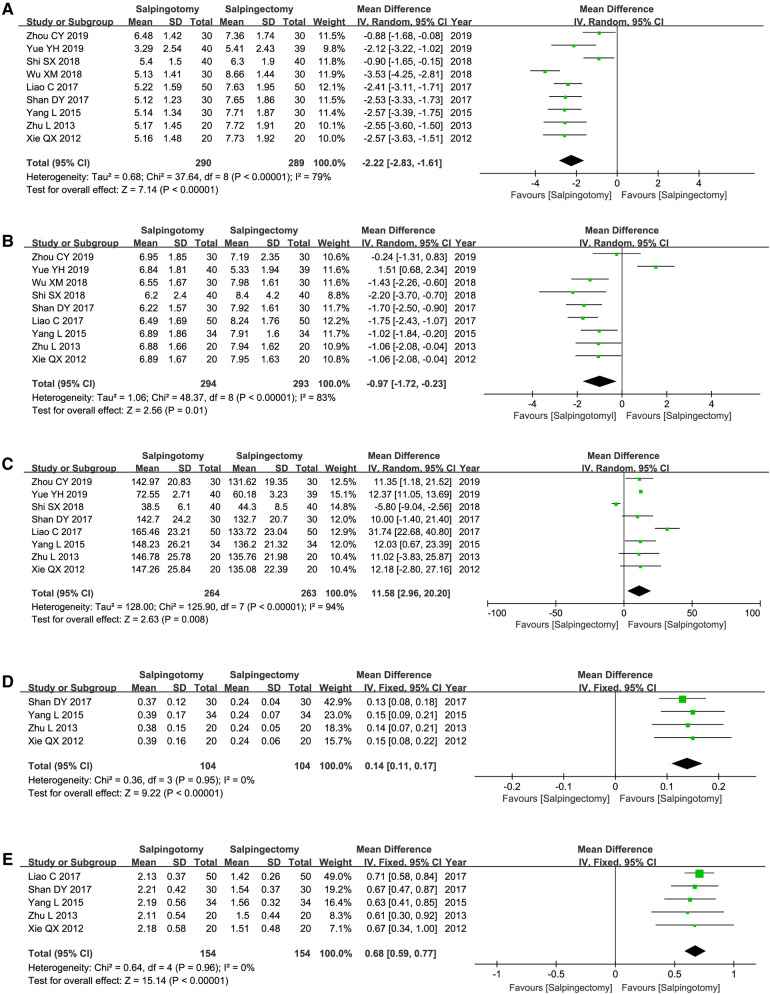
Serum ovarian hormone level. (**A**) the level of FSH; (**B**) the level of LH; (**C**) the level of E2; (**D**) the level of T; (**E**) the level of P.

The values of FSH (WMD = −2.22; 95%CI, −2.8–1.61; *p *< 0.00001) and LH (WMD = −0.97; 95%CI, −1.7–0.23; *p *= 0.01) was signiﬁcantly higher in salpingectomy group. The values of E2 (WMD = 11.58; 95%CI, 2.96–20.20; *p *= 0.008), T (WMD = 0.14; 95%CI, 0.11–0.17; *p *< 0.00001) and P (WMD = 0.68; 95%CI, 0.59–0.77; *p *< 0.00001) was signiﬁcantly higher in salpingotomy group (Figures 5A–E).

#### Ultrasound examination of the affected ovary

**Figure 6:**
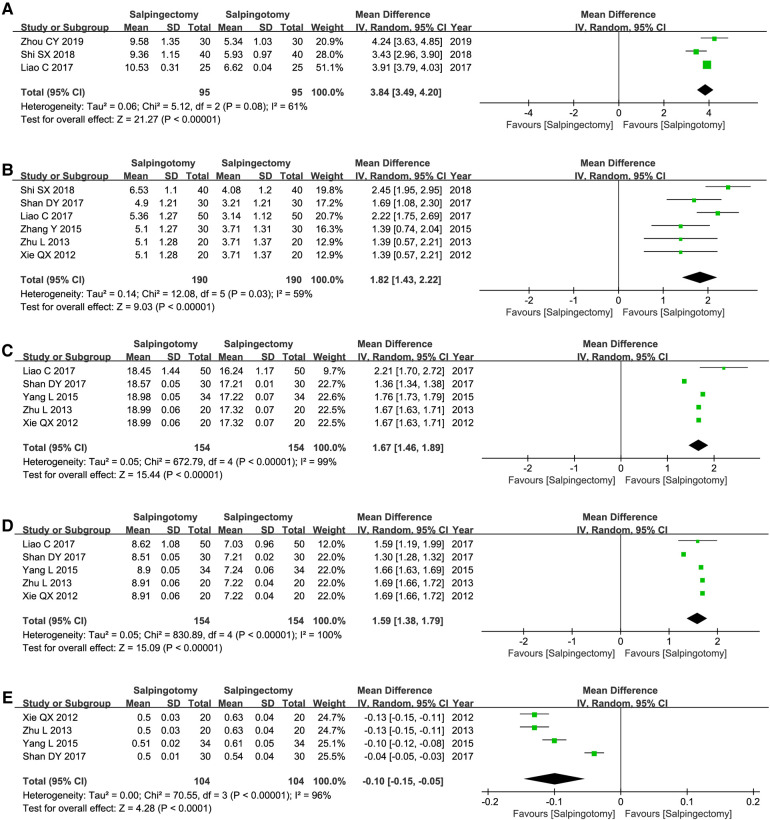
Ultrasound examination of the affected ovary. (**A**) numbers of follicles; (**B**) the cross-sectional area; (**C**) the values of PSV; (**D**) the values of EDV; (**E**) the values of RI.

The numbers of follicles (WMD = 3.84; 95%CI, 3.49–4.20; *p *< 0.00001), cross-sectional area (WMD = 1.82; 95%CI, 1.43–2.22; *p *< 0.00001), PSV (WMD = 1.67; 95%CI, 1.46–1.89; *p *< 0.00001) and EDV (WMD = 1.59; 95%CI, 1.38–1.79; *p *< 0.00001) was signiﬁcantly higher in salpingotomy group. The values of RI (WMD = −0.10; 95%CI, −0.1–0.05; *p *< 0.0001) was signiﬁcantly higher in salpingectomy group (Figures 6A–E).

### Meta-analysis of subgroups

**Figure 7:**
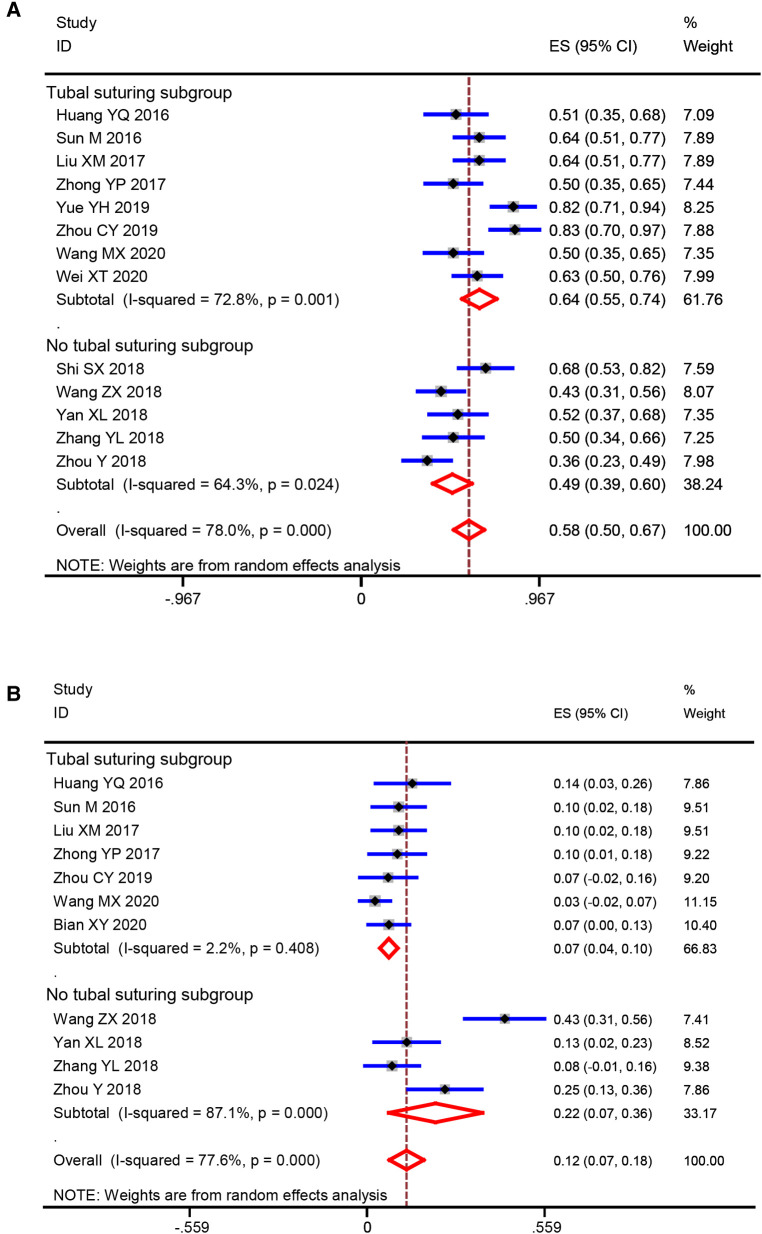
Comparison of salpingotomy with and without suturing (**A**) intrauterine pregnancy rate; (**B**) ectopic pregnancy rate.

At present, whether to suture the tubal after salpingotomy is a controversial clinical issue. In order to clarify whether this factor will affect the results of this meta-analysis, we conducted a subgroup analysis according to with or without suturing. The analysis results were shown in Table 3. Consistently, the results of our subgroup analysis suggested that the postoperative intrauterine pregnancy rate of salpingotomy group is significantly higher than that of the salpingectomy group, regardless of whether with suturing (OR = 4.56, *p *< 0.00001) or not (OR = 1.42, *p *= 0.003). Interestingly, patients with salpingotomy combined with suture showed a significantly lower ectopic pregnancy rate than that of patients with salpingectomy (OR = 2.61, *p *= 0.007). however, salpingotomy without suture did not show this advantage (OR = 2.14, *p *= 0.17). Further analysis found that salpingotomy combined with suture had higher intrauterine pregnancy rate (64% vs. 49%) and lower extrauterine pregnancy rate (7% vs. 22%) than that of salpingotomy without suture (Figure 7).

**Table 3:** Meat-analysis of subgroups according to with or without suturing.

	Subgroup	Salpingotomy (*n*=)	Salpingectomy (*n*=)	Effects model	OR/WMD (95% CI)	*p*-value	Favours
Operating duration	Tubal suturing	231	231	Random	−4.72 (−11.56–2.13)	0.18	–
	No-tubal suturing	114	114	Fixed	−0.04 (−1.29–1.20)	0.95	–
Hemorrhage	Tubal suturing	329	329	Random	307.46 (197.21−417.72)	<0.00001	Salpingotomy
	No-tubal suturing	114	114	Random	152.96 (17.67–288.24)	<0.00001	Salpingotomy
Hospitalize length	Tubal suturing	231	231	Random	−0.48 (−1.42–0.45)	0.31	–
	No-tubal suturing	74	74	Fixed	−0.01 (−0.60–0.58)	0.98	–
β-hCG level	Tubal suturing	217	217	Random	−6.31 (−19.55–6.92)	0.35	–
	No-tubal suturing	74	74	Fixed	−1.23 (−3.17–0.72)	0.22	–
Intrauterine pregnancy	Tubal suturing	341	340	Fixed	4.56 (3.25–6.38)	<0.00001	Salpingotomy
	No-tubal suturing	231	231	Random	1.42 (0.71–2.83)	0.003	Salpingotomy
Ectopic pregnancy	Tubal suturing	305	305	Fixed	2.61 (1.50–4.55)	0.007	Salpingotomy
	No-tubal suturing	191	191	Fixed	2.14 (0.72–6.40)	0.17	–
FSH level	Tubal suturing	120	119	Random	2.00 (1.14–2.87)	<0.00001	Salpingotomy
	No-tubal suturing	170	170	Random	2.39 (1.50–3.27)	<0.0001	Salpingotomy
LH level	Tubal suturing	84	84	Fixed	0.83 (0.28–1.38)	0.003	Salpingotomy
	No-tubal suturing	210	209	Random	1.08 (0.03–2.18)	0.03	Salpingotomy
E2 level	Tubal suturing	124	124	Fixed	12.34 (11.05–13.63)	<0.00001	Salpingotomy
	No-tubal suturing	140	140	Random	11.82 (−8.1–31.75)	0.02	Salpingotomy
T level	Tubal suturing	54	54	Fixed	0.15 (0.10–0.19)	<0.00001	Salpingotomy
	No-tubal suturing	50	50	Fixed	0.14 (0.10–0.17)	<0.00001	Salpingotomy
P level	Tubal suturing	54	54	Fixed	0.62 (0.45–0.80)	<0.00001	Salpingotomy
	No-tubal suturing	100	100	Fixed	0.70 (0.59–0.80)	<0.00001	Salpingotomy
Cross-sectional area	Tubal suturing	50	50	Fixed	1.39 (0.88–1.90)	<0.00001	Salpingotomy
	No-tubal suturing	140	140	Random	2.01 (1.58–2.45)	<0.00001	Salpingotomy
PSV	Tubal suturing	54	54	Random	1.72 (1.63–1.80)	<0.00001	Salpingotomy
	No-tubal suturing	100	100	Random	1.64 (1.35–1.92)	<0.00001	Salpingotomy
EDV	Tubal suturing	54	54	Random	1.67 (1.64–1.70)	<0.00001	Salpingotomy
	No-tubal suturing	100	100	Random	1.52 (1.19–1.85)	<0.00001	Salpingotomy
RI	Tubal suturing	54	54	Random	0.11 (0.08–0.14)	<0.00001	Salpingotomy
	No-tubal suturing	50	50	Random	0.08 (0.00–0.17)	<0.00001	Salpingotomy

## Publication bias

**Figure 8:**
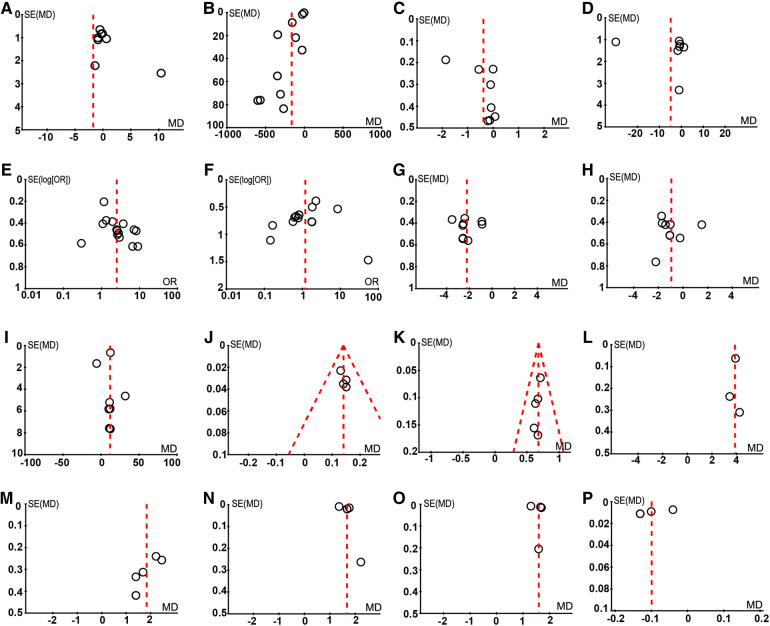
The assessment of publication bias. (**A**) Operating duration; (**B**) The volume of bleeding during operation; (**C**) Postoperative hospitalize length; (**D**) Postoperative hCG level; (**E**) Intrauterine pregnancy; (**F**) Ectopic pregnancy; (**G**) FSH; (**H**) LH; (**I**) E2; (**J**) T; (**K**) P; (**L**) Numbers of follicles; (**M**) Cross-sectional area; (**N**) PSV; (**O**) EDV; (**P**) RI.

Figure 8 demonstrates that the funnel plots have no discernible asymmetry, which indicating a low probability of publication bias.

## Discussion

With the development and improvement in laparoscopic technology, the advantages of laparoscopic surgery in the treatment of tubal pregnancy are being clinically recognized. The incidence of tubal pregnancy is gradually increasing, and the number of patients with reproductive requirements is also increasing. Therefore, protecting the reproductive function of patients after treatment has become a clinical hot spot. Laparoscopic salpingotomy and salpingectomy are common methods for the treatment of ectopic pregnancy. Nonetheless, perioperative safety, postoperative fertility, and the impact on ovarian reserve function of the two surgical methods are still controversial.

Our results indicated that no difference in the operation time and postoperative hospitalization time between the two surgical methods, but the volume of bleeding in patients with laparoscopic salpingotomy and the damage to patients is less than that in salpingectomy. Salpingectomy can completely remove the affected side of the fallopian tube and completely remove the focus. Because salpingotomy retains the diseased fallopian tube, it may increase the risk of embryonic tissue residue. A comparison of the postoperative hCG levels of patients reveals no significant difference in hCG levels between the two surgical methods, suggesting that salpingotomy can also effectively remove the embryonic tissue in the fallopian tube.

Many studies have reported the intrauterine pregnancy rate after ectopic pregnancy surgery, but so far, there is no consensus on the impact of salpingotomy and salpingectomy on fertility in patients with ectopic pregnancy. Although salpingectomy retains the contralateral fallopian tube, it will still affect the postoperative fertility rate of patients. Studies (9, 34–36) have found that if the contralateral fallopian tube is healthy, there is no difference in fertility between salpingotomy and salpingectomy. However, for patients with infertility history, fallopian tube disease, or age more than 35 years old, the intrauterine pregnancy rate after salpingotomy is significantly higher than that of patients with salpingectomy (37–38). Our meta-analysis results also suggest that without considering these confounding factors, the intrauterine pregnancy rate of patients after salpingotomy is significantly higher than that after salpingectomy, and there is no difference in the incidence of ectopic pregnancy. Therefore, the reproductive function of patients is better protected by laparoscopic salpingotomy. Interestingly, subgroup analysis results showed patients with salpingotomy combined with suture exhibited a significantly lower ectopic pregnancy rate than that of patients with salpingectomy. Besides, salpingotomy combined with suture had higher intrauterine pregnancy rate and lower extrauterine pregnancy rate than that of salpingotomy without suture. Therefore, salpingotomy combined with suture should be recommend, according to the results of this meta-analysis. At present, whether to suture the tubal after salpingotomy is a controversial clinical issue, we expect multi-center, large sample randomized controlled studies to further illustrate this problem.

The ovary is an important reproductive and endocrine organ of women, which has the functions of oviposition, ovulation, and endocrine. The blood supply to the fallopian tube and ovary comes from the fallopian tube branches and ovarian branches from the uterine artery and ovarian artery. These branches coincide with each other in the mesosalpinx to form a rich vascular network. The anastomotic arch of the intra mesosalpinx artery is vulnerable to damage during fallopian tube surgery, affecting the blood supply of the ipsilateral ovary (39). Mekin et al. (40) found that after salpingectomy, the average pulsatile index, RI and systolic/diastolic ratio of patients' ovaries were significantly lower than the normal level. Therefore, salpingectomy can easily destroy the blood supply of the ipsilateral ovary. However, the incision of salpingotomy is located on the opposite side of the mesosalpinx, which can preserve the normal function and structure of the fallopian tube, reduce the injury of the mesosalpinx vessels, and preserve the normal blood supply of the ovary. This meta-analysis reveals that the PSV and EDV of the internal stromal artery of the affected side of the ovary in patients with laparoscopic salpingotomy 6 months after operation were significantly higher than those of salpingectomy, suggesting the blood supply of the affected side of the ovary can be better preserved by laparoscopic salpingotomy.

Chan et al. (41) found that laparoscopic salpingectomy on the affected side can block a part of the blood supply to the fallopian tube and ovary, resulting in a decrease in the number of ovarian follicles on the affected side, consequently, a decrease in ovarian reserve function on the affected side. Ovarian volume and the number of sinus follicles can reflect the reserve function of the ovary. The number of sinus follicles is a stage in the growth and development of follicles, which is the precursor of mature follicles. When ovarian function decreases, the number of sinus follicles also shows a parallel downward trend. Similarly, the number of sinus follicles is closely related to ovarian volume. When ovarian reserve function decreases, ovarian volume decreases. Meta-analysis showed a significantly higher number of follicles in the affected sinus and the cross-sectional area of ovaries in patients who underwent salpingotomy than those who underwent salpingectomy, indicating that salpingectomy reduced the reserve function of the affected ovary. In addition, Serum ovarian hormone level, such as FSH, LH, E2, T and P are all sensitive indicators to evaluate ovarian endocrine function. With the decline in ovarian function, FSH and LH levels increase, while E2, T and P levels decrease. This meta-analysis showed that the serum FSH and LH levels of patients with salpingotomy were significantly lower than those in the salpingectomy group 6 months after the operation, and the levels of E2, T and P were significantly higher than that in the salpingectomy group, suggesting better preservation of the ovarian endocrine and reproductive function of patients after that laparoscopic salpingotomy can, which then improves the probability of postoperative second pregnancy.

Pretreatment with methotrexate or mifepristone can effectively inhibit the proliferation of trophoblasts and induce embryonic death, which could reduce the HCG level and intraoperative bleeding (42, 43). However, these ectopic pregnancy patients included in this study were not pretreated with methotrexate or mifepristone before laparoscopic surgery, which exactly will increase the bias of the results, especially for salpingotomy. Besides, a potential limitation of our meta-analysis is that most of the included studies were from China. In fact, we have included two literatures from different countries include 645 patients, mainly from the United Kingdom, Netherlands, United Kingdom, France and United States, accounting for 27.4% (645/2,354) of the total number of patients included in this meta-analysis. However, a potential limitation of this meta-analysis that 72.7% of the included patients were from China, which may affect the representativeness of the conclusion. Another potential limitation is that the surgical experience used by different hospitals, perioperative management methods, and the urgency of patients for fertility may produce different results and increase the heterogeneity of included studies. Therefore, it is necessary to further conduct a well-designed large-scale multicenter randomized controlled trial to study the equivalence or non-inferiority of laparoscopic salpingectomy and salpingotomy in the treatment of tubal pregnancy.

## Conclusion

For patients with tubal pregnancy, the natural intrauterine pregnancy rate after laparoscopic salpingotomy was significantly higher than those who underwent salpingectomy. In addition, laparoscopic salpingotomy can better protect the ovarian reserve function and endocrine function and provide favorable conditions for the second pregnancy. Therefore, patients with ectopic tubal pregnancy should give priority to laparoscopic salpingotomy for embryo extraction, but we still look forward to a multi-center, large sample, long-term follow-up randomized controlled study to provide more reliable clinical evidence.

## Data Availability

The original contributions presented in the study are included in the article/Supplementary Material, further inquiries can be directed to the corresponding author/s.
